# A child diagnosed with severe hemophilia A presenting with nephrotic syndrome: a case report

**DOI:** 10.1186/s13256-023-03941-x

**Published:** 2023-05-20

**Authors:** Janith Chandrakumara, Madushika Wijesundara, Givani Amarakoon

**Affiliations:** Department of Paediatrics, Faculty of Medicine and Allied Sciences, Rajarata University of Sri Lanka, Saliyapura, Anuradhapura, Sri Lanka

**Keywords:** Case report, Hemophilia A, Nephrotic syndrome, Child

## Abstract

**Background:**

Nephrotic syndrome occurring as a complication of immune tolerance therapy for inhibitors in hemophilia B is well recognized. It is also known to occur in association with factor borne infections, especially hepatitis C. This is the first case report of nephrotic syndrome occurring in a child receiving prophylactic factor VIII in the absence of inhibitors of hepatitis infection. However, the pathophysiology of this phenomenon is poorly understood.

**Case presentation:**

A 7-year Sri Lankan boy diagnosed with severe hemophilia A on weekly factor VIII prophylaxis was diagnosed with three episodes of nephrotic syndrome, a condition in which there is leakage of plasma protein into urine. He had three episodes of nephrotic syndrome, all of which responded well to 60 mg/m^2^ daily dose of oral steroids, achieving remission within 2 weeks of starting daily prednisolone. He has not developed inhibitors for factor VIII. His hepatitis screening remained negative.

**Conclusions:**

There is a possible link between factor therapy for hemophilia A and nephrotic syndrome, which can be a T-cell-mediated immune response. This case also highlights the importance of monitoring for renal involvement in patients treated with factor replacement.

## Background

Hemophilia is an X-linked inherited bleeding disorder of deficiency of either clotting factors VIII or IX. Hemophilia A occurs in 1 out of 5000 males, and is the most common type of bleeding disorder [1]. Severity of hemophilia is classified according to the plasma factor levels. Mild, moderate, and severe hemophilia are denoted by factor levels of 5–40%, 1–5%, and less than 1%, respectively [2].

Factor replacement is the mainstay of managing patients with hemophilia, though inhibitor development may be challenging to manage [1]. A total of 25–30% of patients with hemophilia A and 2–5% of patients with type B develop inhibitors during factor therapy [3, 4]. The treatment strategy in practice is immune tolerance induction (ITI) to eradicate inhibitors [1, 3, 5].

Nephrotic syndrome is a glomerular disease characterized by proteinuria, hypoalbuminemia, and edema. Proteinuria is defined as urine protein > 40 mg/m^2^ per hour or urine protein:creatinine ratio ≥ 2000 mg/g (≥ 200 mg/mmol) or > 3+ proteinuria on dipstick. Hypoalbuminemia is defined as serum albumin < 25 g/L [6]. Most common glomerular pathology associated with childhood nephrotic syndrome is minimal change disease [6]. The pathophysiology of nephrotic syndrome is complex and not fully understood. There are multiple genetic, circulatory, and immune-mediated factors implicated in its pathogenesis [6]. Nephrotic syndrome is reported to occur in children with hemophilia with inhibitors, immune tolerant treatment for inhibitors, and as a complication of viral hepatitis resulting from factor replacement [7–9]. Here we present a case where nephrotic syndrome occurred in a child with severe hemophilia A who neither had inhibitors nor hepatitis infection.

## Case presentation

A Sri Lankan boy, who is a known patient with severe hemophilia A on weekly factor VIII prophylaxis, presented with two episodes of steroid sensitive nephrotic syndrome. He was diagnosed with hemophilia at 9 months of age, following multiple bruising occurring while playing. His initial clotting profile revealed prothrombin time of 10.7 seconds (10–13), activated partial thromboplastin time of 45.8 seconds (25–36), thrombin time of 16.2 seconds (15–19), and bleeding time using ear lobe method 3 minutes (1–4). His factor VIII levels were 0.34% (50–100%), which confirmed a diagnosis of severe hemophilia A. He started on weekly prophylaxis with recombinant human factor VIII at 4 years of age. He developed an urticarial rash following the administration of factor VIII on two occasions; when he was 5 years and 4 months of age. On the first occasion the child developed urticarial rash involving face and trunk and generalized body itching a few minutes after administration of factor VIII concentrate from pooled human plasma (Koate-DVI, Grifols Therapeutics Inc, USA). He developed a similar urticarial rash a week later when he was administered recombinant factor VIII (HEMOFIL M, Baxter Inc, USA). In both episodes he received a single dose of oral chlorpheniramine, and the symptoms resolved within an hour. There were no breathing difficulties or hypotension to indicate anaphylaxis during either of the episodes. He continued to receive prophylaxis with both these factor VIII preparations without developing any subsequent allergic reactions. He did not have any other atopic features such as food allergies, allergic rhinitis, eczema, or asthma.

During the first episode of nephrotic syndrome that occurred at 6 years of age (following 16 months of regular factor VIII therapy), he presented with a 2-day history of periorbital swelling. The urine dip stick test showed 4+ of albuminuria. His serum albumin level was 23.6 g/L (34–54) and total cholesterol level was 757 mg/dL (< 200). His creatinine was 31 µmol/L (26–62) and blood urea was 2.6 mmol/L (2.1–8.5).

He was started on 60 mg/m^2^/day of prednisolone per KDIGO (Kidney Disease Improving Global Outcomes) guidelines, and he achieved remission (defined as urine albumin trace or nil on urine dip test for 3 consecutive days) on day 13 of treatment. After a total of 28 days of prednisolone at the same dose, it was reduced to 40 mg/m^2^ per alternate day and stopped. The child received inward care for 21 days during this episode.

He remained in remission for 15 months before presenting with the first relapse of nephrotic syndrome. He presented at 7 years of age with periorbital swelling; clinically detected free fluids in the abdomen indicated by horseshoe dullness in the abdomen. He had proteinuria detected by urine strip test 3+ or more for 3 consecutive days. His systolic blood pressure was between the 95th and 99th centiles on presentation, which normalized during the next few days without antihypertensives. His serum albumin level was 12.3 g/L (35–520). His renal functions were normal with creatinine of 44 µmol/L (26–62) and blood urea of 3 mmol/L (2.1–8.5). During the acute period of the illness, he received an albumin transfusion with 5 mg of intravenous furosemide given mid-transfusion. He achieved remission after 12 days of 60 mg/m^2^ per day of prednisolone, it was switched to 40 mg/m^2^ per alternate day and gradually tapered off by 5 mg decrements over 18 weeks. He was hospitalized for 9 days during this episode.

He developed his second relapse of nephrotic syndrome at 7 years and 7 months of age. In this relapse he presented with periorbital swelling and 3+ or more proteinuria on urine dip sticks for 4 consecutive days. His serum albumin was 19.9 g/L (35–52). His renal functions remained within normal limits with creatinine of 33 µmol/L (26–62) and blood urea of 2.9 mmol/L (2.1–8.5). Oral prednisolone 60 mg/m^2^ per day was given for 8 days until he had trace or nil proteins in urine dip stick test for 3 consecutive days. His prednisolone dose was then reduced to 40 mg/m^2^ per alternate day and gradually tapered off by 5 mg decrements over 18 weeks. He received 8 days of inward care during this episode.

His hepatitis virology was negative. Inhibitors screening via incubated mixing test was negative throughout the illness. Renal biopsy or the second-line investigations were not performed as he did not fulfill the criteria in the Sri Lankan national and KDIGO guidelines.

## Discussion

The occurrence of nephrotic syndrome in patients receiving immune tolerant induction (ITI) for inhibitors in hemophilia B is well reported in the literature. These patients have received high doses of factor IX. Some of them have received other immunomodulatory therapy such as cyclophosphamide and mycophenolate mofetil to bring about immune tolerance [3, 9–11].

Although ITI is utilized as a treatment modality of hemophilia A, there are no reported cases of nephrotic syndrome occurring in such patients. Ewenstein *et al*. postulates that this might be related to factor IX being distributed in both intravascular and extravascular compartments, in contrast to factor VIII, which is confined to intravascular compartment. Another hypothesis is that it might be related to the fact that a lesser amount of protein is administered in ITI for factor VIII compared with that of factor IX [3].

Priya Sharma *et al*. reported a hemophilia A patient with inhibitors developing nephrotic syndrome without ITI [8]. Like our patient, this child also had severe hemophilia with factor levels < 1%. He developed inhibitors 6 months after starting factor VIII prophylaxis. He developed nephrotic syndrome at 22 months of age, 10 months since starting prophylaxis. This is in contrast to our patient, who had the first episode of nephrotic syndrome 3 years after starting factor prophylaxis and has not developed inhibitors so far. Similar to our patient, this patient also had steroid-sensitive nephrotic syndrome, achieving remission after 14 days of prednisolone. There was no information about this patient having any allergic reactions.

Another case report describes occurrence of nephrotic syndrome in a 3-year-old with hemophilia A with inhibitors [7]. This child was also having chronic active hepatitis B. In contrast, our patient had neither inhibitors nor hepatitis infection. In this child, development of nephrotic syndrome coincides with detection of inhibitors. The authors hypothesize that nephrotic syndrome might have occurred due to factor VIII immunocomplexes, while the reticuloendothelial system is already saturated with hepatitis immunocomplexes. Clinical picture of nephrotic syndrome is well described in association with hepatitis B [12], however, it is more commonly associated with non-minimal change etiologies that are less responsive to steroids [13]. Unfortunately, this case report did not mention renal biopsy findings or steroid responsiveness.

The only other case report in the literature that describes hemophilia associated with nephrotic syndrome is in a 74-year-old man from Japan [14]. This case report describes acquired hemophilia A following a relapse of minimal change nephrotic syndrome. Acquired hemophilia, an extremely rare condition, has a different pathophysiology to congenital hemophilia. It occurs due to the development of antibodies (inhibitors) against factor VIII [15]. This case is significantly contrasts to our case as this describes an adult who had nephrotic syndrome long term and went on to develop antibodies against factor VIII.

In our patient, nephrotic syndrome occurred in the absence of inhibitors or hepatitis infection. He was successfully treated with oral prednisolone during three episodes, which indicates he most likely had minimal change disease.

It is unclear whether the patient having episodes of urticaria is related to the occurrence of nephrotic syndrome. Minimal change disease is known to be associated with allergies and atopies [16, 17]. However, lack of any other features of atopy and the significant time gap between the urticarial reactions and the onset of nephrotic syndrome make it an unlikely association in our opinion.

To the best of our knowledge, this is the first case report of hemophilia developing nephrotic syndrome in the absence of inhibitors or hepatitis infection. It is worth noting that both inhibitors and hepatitis causing nephrotic syndrome are most likely immune complex mediated. Immune-complex-mediated nephrotic syndrome tends to manifest as non-minimal change disease, whereas minimal change disease is thought to be due to a T-cell-mediated immune response [18]. T-cell response to factor VIII is identified and postulated as a possible reason for development of inhibitors in patients treated with factor VIII [19]. A possible explanation for the development of nephrotic syndrome in patients treated with factor VIII is T-cell-mediated immune response triggering a microstructural change in the podocytes. This would explain why all described cases of this nature occurred in patients receiving factor VIII prophylaxis.

However, with our current knowledge, it is not possible to confirm whether occurrence of these two disease entities is associative or coincidental.

Discussion with the mother revealed that medical follow-up for two chronic conditions caused economic and social strain on the family. Furthermore, she expressed that she is worried about lack of an explanation as to why both conditions affected her son.

## Conclusions

There is a possible link between factor therapy for hemophilia A and nephrotic syndrome that can be a T-cell-mediated immune response. This case also highlights the importance of monitoring for renal involvement in patients treated with factor replacement.

## Data Availability

Data sharing is not applicable to this manuscript as no datasets were generated or analyzed.

## Abbreviation

ITI: Immune tolerance induction
